# Codepoietic Generation of Meaningful Information in the Evolving Biosphere

**DOI:** 10.3390/e27070672

**Published:** 2025-06-24

**Authors:** Abir U. Igamberdiev

**Affiliations:** Department of Biology, Memorial University of Newfoundland, St John’s, NL A1C 5S7, Canada; igamberdiev@mun.ca

**Keywords:** codepoiesis, complexification, knowledge acquisition, evolution, meaningful information, sustainable non-equilibrium

## Abstract

Meaningful information represents reality in its potential form, and its actualization increases the system’s negentropy. Biological evolution leads to the expansion of meaningful information by generating new coding systems (codepoiesis). Through this expansion, any evolutionary change obtains functional value when it receives an interpretation through which it gives rise to a meaningful function. Complexification in the evolutionary process corresponds to the generation of new meaningful information and, thus, to the development of new structures with corresponding functions. Any biological function has a meaning within the context of a particular environment, and the evolutionary search for new meanings results in the establishment of the state of sustainable non-equilibrium acting as an attractor, in which the developing system achieves the condition of maximization of its power via synergistic effects. At higher levels of the organization, evolutionary innovations emerge as niche constructions, behavioral choices, and, finally, the phenomenon of cognition. The evolutionary growth of meanings appears as a part of the expanding information system formed by the organisms inhabiting it. It acquires major expansion with the emergence of consciousness that incorporates the image of the whole world into the dynamic process of knowledge acquisition and creates the conditions for the development of global civilization.

## 1. Introduction: Meaningful Information and Its Growth

The evolutionary growth of biological systems corresponds to an increase in the meaningful information in them. Brenner and Igamberdiev [1,2] introduced the definition of meaningful information as a reality in potential form. This definition arises from Aristotle [3], who distinguished between the *dynamis* corresponding to the potentiality of the physical state, the *episteme* corresponding to the information that is inherited and can be transferred, and the *theorein* corresponding to the actual workout and implementation of the information. In fact, Aristotle anticipated the understanding of biological inheritance as a transfer of genetic information [4].

The nature of an internal engine for the generation of information as a reality in its potential form in biological systems was partially clarified in the last century; however, many aspects of its emergence in the course of evolution require further clarification. In fact, the actualization of meaningful information leads to an increase in the system’s negentropy, and this occurs through the establishment and growth of coding relations representing the internal description of a biological system. The entailing of meaning in the information can be illustrated by the following example: e.g., for a prey, the information received as the smell of a predator is the predator in potential form. The prey, having interpreted these signs, undertakes actions to ensure this potentiality does not become actualized [1]. However, these actions are possible if they are memorized in the digital structures of memory (internal description) and in some modes of instinctive behavior that can be inherited. The meaningful specification representing the qualitative aspect of information relates in biological systems to the quantitative aspect of information as a digital internal description, and thus, the actualization of meaningful information tends to increase the order, i.e., the system’s negentropy.

The idea of a biological system possessing an internal description became evident from the discovery of Mendel’s laws. It received real interpretation in molecular biology in the concept of matrix coding, which was anticipated by Nikolai Koltsov (Koltzoff) in 1927 [5,6] and formulated by George Gamow in 1954 [7]. The genetic code was deciphered by Nirenberg and Matthaei [8] in 1961, following the innovative experimental strategy of Crick, Barnett, Brenner, and Watts-Tobin [9]. As a result, it was established that the genetic code is a triplet code, it is degenerate, triplets are not overlapping without commas (although introns were discovered later), and each nucleotide sequence is read from a specific starting point [10].

The systems having the internal description must include the mechanisms of generation of new, meaningful information [11]. According to the cited paper, the incompleteness of formal description possessed by the systems defined as epigenetic (because they have properties that go beyond genetic information) results in the potential assignment of new values to the statements of formal systems that previously were not defined. This process of Gödel numbering introduces a new coding to the system, which overcomes the undecidability of the initial statements. The undecidable statements become the basis of the evolutionary expansion of the system by acquiring new meanings. The complexification process taken in the framework of formal logic represents a relative resolution of the paradox of undecidability via assigning new coding elements (Gödel numbers) to the undecidable statements, which results in signifying them and assigning the values (meanings) to them. Logically, this process corresponds to the introduction of a new code that signifies the statements that were previously undefined [12]. The concept of growing the coding systems via the generation of hypertextual statements corresponds to a generation of new meaning for the existing structure that leads to its evolutionary transformation. This denotes the emergence of new significations in the coding systems, defined by Marcello Barbieri [13,14] as a *codepoiesis* (Box 1). Two and a half thousand years ago, Heraclitus named this process a *Self-growing Logos*.

Box 1 **Codepoiesis as a driver of evolutionary change.**

Incomplete analog identification of the externality → Undecidability in the internal digital system → Codification via generation of Gödel numbers → New meaningful information → New biological organization

## 2. RNA as a Mediator of the Codepoiesis

The understanding of the role of RNA as a mediator in the realization of meaningful information contained in DNA, which is the *episteme* in Aristotelian terms and corresponds to the genotype, to form its actual realization, the *theorein*, as defined by Aristotle, corresponding to the dynamic phenotype, was the main breakthrough made by the invention of molecular biology. The decoding of meaningful information from the genotype occurs via the joint action of messenger, transfer, and ribosomal RNAs. This discovery became the basis for the formulation of the central dogma of molecular biology [15]. The changes in genotype that lead to evolutionary transformations were still considered fully spontaneous and random, although the evidence accumulated that the combinatorial processes leading to genome transformations have their constraints and can be mediated by the pool of macromolecules that direct the processes underlying genetic changes. The discovery of mobile genetic elements [16], which could also cause disturbance at the stage of meiosis, became an important step in the understanding of constraints of evolutionary rearrangements. Later, more knowledge was accumulated, revealing the specific roles played by different types of non-coding RNAs in governing genome regulation and its rearrangements [17].

The discovery of microRNAs [18,19] revealed the ancient mechanism of gene regulation based on RNA interference with gene expression. Although such a mechanism exists at the level of prokaryotes, true microRNAs appear only at the level of eukaryotes [20]. Other types of RNA can directly participate and control the combinatorial events occurring at the levels of genomes and messenger RNA processing (splicing). The types of non-coding RNAs include microRNA (miRNA) and small interfering RNA (siRNA), long non-coding RNA (lncRNA) and enhancer RNA, double-stranded RNA (dsRNA), and circular RNA (circRNA). Specific types of RNA include CRISPR RNA in prokaryotes, whose function is to edit DNA in situ to provide protection against virus invaders.

To understand the evolutionary process, Ervin Bauer [21] in 1935 suggested the idea of the intensification of the process of the search for new genetic variants to overcome the limits of adaptation. This received support at the physicochemical level with the discovery of mobile genetic elements and became the basis of modern evolutionary concepts. These concepts include the idea of an environmentally regulated meiotic crossing-over, or eco-crossover, which determines the genetic diversity of eukaryotes [22], the model of evolution based on the rearrangement and growth of the program of differentiation that underlies biological morphogenesis [23], and modern epigenetic concepts of evolution [24,25]. According to Olovnikov [22], meiotic recombination generates epimutations, i.e., eco-dependently marked chromosomal sites that can be transformed into mutations. He suggests that eco-crossover uses the eco-stress-dependent versions of circular RNAs, which are synthesized as variants of alternative splicing. These circular RNAs, binding to homologous epimutations on the homologous parent chromosomes of the meiocyte, involve them in topologically specific recombinations that further create random mutations in nonrandom genomic sites. These quasi-random mutations serve as a pivotal source for the emergence of all adaptations of any level of complexity [22]. Recombination at the level of nucleic acids “overcomes the cumbersome task of data management” [26]. There should be specific mechanisms that generate epimutations upon the approximation of the signal transduction system to its limits. The limits of adaptation framed within the analogous semiotic system trigger the mechanisms of their expansion via the evolutionary generation of new coding systems. The circular RNA mechanism remains hypothetical, but it provides a possible solution for understanding the evolutionary expansion of digital information in biological systems (Box 2).

Box 2 **Generation of epimutations and mutations in meiotic recombination.**

Ecological stress: Approaching adaptability limits → Expression of circular RNAs → Epimutations → Meiotic eco-crossover → Novel genetic combinations/mutations → Codepoiesis → Selection of useful outputs

## 3. Codepoietic Generation of Meaningful Information in Autopoietic Systems at the Level of Funktionskreis

The basic self-supporting autopoietic organization (self-contained space) of biological systems assumes the structure in which catalysts also realize their own self-reproduction. Tibor Gánti originally outlined this structure in 1952, presented in more detail in Hungarian in the 1960s [27], and published in English many years later [28]. Other approaches to autopoietic structures include the concept of Humberto Maturana and Francisco Varela, who suggested the term autopoiesis [29], Robert Rosen’s model of (M,R) systems [30], the concept of autocatalytic sets of proteins of Stuart Kauffman [31], and the hypercycle concept of Manfred Eigen and Peter Schuster [32]. Freeman Dyson [33] outlined similar ideas in his approach to the origin of life, and in a general representation, they can be traced to Schrödinger [34].

Although the autopoietic structure describes, in general, the self-maintenance of a living system, it should be flexible to adapt to changing environmental conditions. This is achieved via the functional circle (or cycle) of adaptation originally introduced by Jakob von Uexküll in 1926 as the *Funktionskreis* [35]. Uexküll anticipated the notion of the algorithm in the operation of the functional cycle and made a guess about molecular hereditary structures. While in the autopoietic structure, the meaning appears internally, in the *Funktionskreis*, the meaning is attributed to the external events appearing as the signs in the Umwelt, which is “inhabited” by the living system. The sensing of the external biosystem events in the Umwelt and reacting to them assumes the regulation of the system at different levels. It includes the feedback from the phenotypic response to the level of genotype, which is achieved through the activation or suppression of transcription and translation. The classes of regulatory RNA, such as miRNAs, play this role in the course of operation of the *Funktionskreis*, which represents the first reflexive cycle that provides the adaptive fitness of the autopoietic system. James Barham [36], who suggested a physical theory of the meaning of information, further developed this concept. He identified biological functions with generalized non-linear oscillators and their associated phase-space attractors and then postulated that such oscillators contain a component capable of coordinating low-energy interactions with the correct environmental conditions supporting the dynamical stability of the oscillator. In this concept, the meaning of information is interpreted as the prediction of successful functional action.

The fundamental challenge of biological evolution consists of the connection of the codepoiesis with the semiotic ability, granting the system the property of expansion and following memorization of the function circle proposed by Uexküll. In the evolution of life, autopoietic systems possess signal-processing semiotic subsystems, which include sensors capable of detecting specific changes in the external world and transforming them via signal transduction within the structure of the functional circle. Thus, the *Funktionskreis* involves semiosis based on analog information processing, which in turn relates to the digital coding system as a component. Codepoiesis emerges through the crosstalk between the analog and digital components of the autopoietic system in the course of its continuous interaction with the environment.

It is important to note that such an autopoietic system, when it reaches the limits of adaptation, can generate the codepoietic events to overcome these limits in the course of evolution. The programmed codepoietic events of such type are related to reproduction. However, the alterations of genetic material in the course of reproduction or between the reproduction cycles cannot be reduced only to random mutations. Already at the level of prokaryotes, in particular, CRISPR RNAs that are able to edit DNA in the course of protection against invading external genetic elements can directly affect the evolutionary process [37]. In the simplest autopoietic systems, the evolution of ribozymes follows from the dual function of nucleotides serving both as the energy currency molecules of the general metabolism as free nucleotides and participating in the transfer of genetic information via covalent polymerization to nucleic acids [38]. This dual function of nucleotides (their energetic and coding capacities), starting from the putative stage of life origin representing the RNA world, determines that environmental changes influence both energetic metabolism and the processing of nucleic acids. Any changes triggered by environmental constraints directly affect both bioenergetic pathways and nucleic acid turnover, resulting in the switches in their operation and leading to rearrangements of the autopoietic structure. The relative weight of the energetic versus the informational component may be considered the original basic trigger for codepoiesis when the system approaches the limits of its autopoietic performance.

## 4. Origin of Eukaryotes and Novel Realizations of the Codepoiesis

The expansion of the set of biological codes associated with the appearance and complexification of eukaryotic cells (eukaryogenesis) and the evolution of multicellularity remains the most important challenge for modern biology. We can formulate a general statement that the complexification of the eukaryotic structure and further multicellularity is based on the development of higher codes operating over the genetic system. The evolutionary process involves the realization of the bipolar choice, e.g., between unikonts and bikonts, in the divergence between protostomes and deuterostomes, and in all events of cellular differentiation manifested as differentiation trees. In all these events, the internally controlled recombination process, in particular, in the course of meiotic cell division and ontogenetic differentiation, becomes the main driving factor. It corresponded to the evolution of eukaryotes and multicellular organisms and the increasing role of the epigenome and epigenetic regulation in the complexification of biological organization. Evolutionary complexification unfolds as a propagating non-deducible construction following the generation of functional redundancy, which is achieved through gene duplication, symbiosis, cell–cell interactions, etc., and becomes an important prerequisite for the appearance of new evolutionary meanings.

Eukaryotes developed a powerful combinatorial system based on splicing. The splicing process significantly increases the combinatorial power of the evolving biological systems [39] and, thus, the generation of new evolutionary meanings. Although the splicing process was widespread in Archaea, which became the predecessors of eukaryotic cells, its increased flexibility and complexity were fully realized with the appearance of eukaryotes. The evolution toward the appearance of the first eukaryotic system was slow, and the evolution toward complex multicellularity continued for more than a billion years (over the Proterozoic eon). The rate of this evolution, in particular, was limited by the release of ions in the Proterozoic oceans that triggered the formation of the enzymes participating in the cross-linking of cells in the multicellular organism, including copper-containing oxidases that are active at lower pH values [40]. The complex organization of eukaryotic cells, possessing an increased combinatorial flexibility of the genetic material, significantly increased the generation of meanings in the biosemiosphere formed by eukaryotic organisms.

The origin of eukaryotic cells is related to the attainment of the mitochondrion, serving as an energy-generating organelle. Bioenergetic limitations constrained the genome size in prokaryotes, while a manifold expansion of the number of expressed genes in eukaryotes was directly linked to the acquisition of mitochondria [41]. A substantial increase in genomic capacity made the complexification of eukaryotic structures and multicellularity possible via the use of mitochondrial power [42,43]. The dual function of nucleotides as energy molecules and as the components of nucleic acids determines the balance between metabolism and repair (or replacement) of the elements in the autopoietic structure in Robert Rosen’s sense [30,38,44]. Active generation and transport of the macromeric compounds by mitochondria open the possibilities of active rearrangements of genetic structures in the course of splicing, recombination, and horizontal gene transfer.

Eukaryotic cells have a much higher capacity for the generation of new meanings via the interactions of the nuclear and organellar (mitochondrial and chloroplastic) genomes, the advancement and complexification of the splicing process, etc. There are different arrangements of the codepoietic process in different groups of eukaryotes. One type of arrangement was realized in ciliates, which perform the spatial separation of the generative and vegetative functions via the two different sorts of nuclei [17]. A diploid micronucleus carries the generative function but does not express its genes, while the ampliploid macronucleus participates in the expression of the phenotype via general cell regulation, in particular, by providing the small nuclear RNA for vegetative growth. The macronucleus originates from the micronucleus by amplification of the genome and heavy editing, which occurs after hundreds of generations when the macronucleus shows signs of aging [45]. Epigenetic regulation of DNA rearrangements in ciliates is realized via RNA-directed DNA editing, in which the non-coding RNAs participate [17]. While ciliates possess the spatial separation between the generative and vegetative functions, in all other groups of eukaryotic organisms, these functions are temporally separated and belong to the same nucleus.

The organization of combinatorial events in eukaryotic cells resulted in the appearance of a huge number of new evolutionary inventions related to the appearance of novel meanings. Many of them were related to endosymbiotic events, with the shuffling between the nuclear and organellar genomes. This includes the origin of mitochondria determining the energetic power and autonomy of the eukaryotic cell [42], the appearance of different types of chloroplasts varying in the phylogenetic groups of Chromista [46], and the emergence of the eukaryotic organelle nitroplast fixing atmospheric nitrogen in some coccolithophores [47]. There are several modifications of the symbiotic organelles corresponding to different evolutionary purposes and meanings. Algal chloroplasts evolved multiple times through secondary and tertiary endosymbiosis [48]. Hydrogenosomes represent a modification of mitochondria to anaerobic environments in some anaerobic ciliates, flagellates, and fungi, where they produce molecular hydrogen and ATP in anaerobic conditions [49]. Numerous taxonomic groups of Protista and Chromista having polyphyletic origin appeared through the variations of different types of endosymbiotic events, followed by complex rearrangements of the nuclear and organellar genomes.

An increase in the capacity for combinatorial rearrangements and thus the generation of new semiotic, communicative, and meaning-bearing features of the biological systems and processes is reflected in the expansion of the splicing processes not only in the nuclear genomes but also in the organellar genomes, which originally did not have introns and splicing mechanisms. Organellar introns encode many proteins, including maturases, homing endonucleases, reverse transcriptases, and some ribosomal proteins, and contain novel open reading frames [50]. This significantly increases the combinatorial capacity of eukaryotic organisms and preconditions the appearance of multicellular organisms with highly differentiated types of cells forming different tissues.

## 5. Complexification of Multicellular Organisms: The Second Functional Cycle

The structure of the functional cycle (*Funktionskreis* in Uexküll’s concept) includes the sequences of the reactions of perception and action, in which a primal awareness of the environmental condition leads to the adaptive behavior of the perceiving agent. In this sense, the functional cycle continuously generates meanings at the interface between the organism and its environment. The operation of the functional cycle results in the adaptive adjustment of metabolic processes to the changes in the environment. This adjustment is realized in animals, in particular, through the nervous system, whose operation is not limited by the perception of external signals and response to them but also includes the emerging hierarchically controlled features, such as the reflection of this perception, which becomes a basis for more complex behavior. It is realized via the second functional cycle (or rather the set of cycles), corresponding, in particular, to behavioral instincts. We can claim that the behavior of animals possessing a sufficiently developed nervous system is structurally organized into two interconnecting cycles, one of which perceives external signals, and the other perceives the operation of the first cycle [51]. With the emergence of the central nervous system (CNS) and the brain structure, the whole system perceives, in addition to the external signal, the functional cycle corresponding to its perception. The first loop is able to perceive low-energy external signals, e.g., corresponding to single photons by the adapted eye, which was demonstrated experimentally [52], and the second loop should perceive and recognize low-energy dissipation from the first loop. This conclusion is based on theoretical considerations of the operation of the nervous system [51] and requires solid substantiation in the experiments. The basis of such substantiation could be the concept of the morphogenetic field formed by ultra-weak photon emissions in biological systems, as suggested by Alexander Gurwitsch in the early 20th century [53]. Very low radiation from single cells, which is generally lower than 10^−20^ W, was experimentally detected [54]. Recent experiments revealing that the brain governs morphogenesis in animals [55,56] can be considered as indirect supporting evidence for the hypothesis of the implementation of the second loop of control over the functional cycle through the central nervous system.

According to Aristotle, the sensitive–locomotive “animal soul” is distinct from the nutritive “plant soul”. Although only arthropods, cephalopods, and vertebrates have a true brain, its developed precursor structures exist in lower organisms except sponges and diploblasts [57]. The sensitive–locomotive recognition loop senses the initial perceptive functional cycle and controls its activity via the set of specific internal receptors. In plants, certain structures resemble the recognition loop of the second cycle, which includes the actin-based domains for cell–cell communications and other integrated networks [58]. This becomes the basis of their purposeful actions during adaptation, which do not reach the complexity level of animal self-actions [59].

The loops of perception (the first functional cycle) and recognition of perception (the second functional cycle) are based on the complex cytoskeletal structures. The cytoskeleton is the macromolecular system that expands the function of conformational relaxation of biological molecules to the cellular and multicellular levels. At the level of individual enzyme molecules, the conformational relaxation is responsible for the functions of catalysis and molecular transport, while the conformational relaxation of the cytoskeletal units performs such macroscopic functions as the replication of nucleic acids during cell division, cell contraction, flagella movement, and the coordinated transformation of perceptive signals and organismal reactions to them. All macroscopic functions of complex organisms are governed by cytoskeletal structures. The emission of photons of ultra-weak energy during conformational relaxation of cytoskeletal fibrils could be the basis of the arrangement of functional cycles into the integrated structure of the multicellular organism [60,61]. The problem of internal reflexive perception and its receptors, in addition to the perception of external signals, may become the main challenge for the further development of biology.

## 6. Morphogenesis of Multicellular Organisms as a Realization of Meaningful Information

The process of morphogenesis of the multicellular organism attains different meanings during cell division as the cells form different tissues. Despite the fact that all cells contain the same genetic information, they express it in different ways. This corresponds to the huge expansion of reality in potential form, representing meaningful information with the appearance of multicellular organisms. The idea that the process of morphogenesis has much in common with perception arises from Edmund Sinnott [62] (1961). From this point of view, we can analyze morphogenesis as the process of a higher hierarchical level than the basic genetic system of the organism, which is based on distinguishing and realizing alternative possibilities existing in the genetic system. This occurs through the arrangement of hyper-restorative dynamics [63] that can be considered as an expansion of the principle of a sustainable non-equilibrium state [21]. While the sustainable non-equilibrium state determines the maintenance of the autopoietic homeostatic structure of the cell and organism [38,64], the hyper-restorative non-equilibrium state forms the background for the complexifying homeorhetic organization of the developing organism [65]. In this self-growing dynamic organization, novel morphogenetic structures are formed [66].

The expansion of reality in its potential form is based on the increased combinatorial power of the genome, but it is governed by hierarchical processes of a higher level, which is in a general representation described in Waddington’s concept of epigenetic landscape [65,67]. In forming the epigenetic landscape, the governing role belongs to the processes occurring through the cytoskeletal dynamics. Hyper-restoration as the basis for morphogenesis occurs at the level of the cytoskeleton [63]. The cytoskeleton, in the course of hyper-restorative dynamics, becomes the medium for penetrating differentiation waves, determining the fate of cells in the developing organism [23], and it also emits low-energy quanta, which can be perceived and interpreted within the whole multicellular organism according to the ideas introduced by Gurwitsch [53]. Volodyaev et al. [61] presented the recent developments of Gurwitsch’s ideas on ultra-weak emissions in biological systems.

While the enzymes performing the transfer of chemical structures, elementary particles (electrons and protons), or catalyzing biosynthetic reactions require nucleotide coenzymes as the tools for electron, molecular, or radical transfer, the cytoskeletal components also require the nucleotide constituents for their conformational dynamics. While ATP is consumed for actin and myosin contraction, other nucleotide structures participate in transforming the signals in the course of cytoskeletal action. The governing of the transcription process by the cytoskeleton was demonstrated by introducing fluorescently labeled pre-miRNA into living cells. The experiments showed the directed movements of miRNA on microtubules and actin filaments, realizing the cytoskeleton-dependent miRNA trafficking that leads to miRNA association with the nucleus and the endoplasmic reticulum/Golgi apparatus and results in the dynamic interaction of miRNA with cellular organelles regulating transcription [68]. The association of the cytoskeleton to the endoplasmic reticulum and Golgi also generates the dynamic force for membrane movement, resulting in the translocation of the organelle or deformation of its membrane, which, in particular, is very common in such polarized cells as neurons [69].

We still know very little about the coding systems determining multicellularity. Morphogenesis can be mapped into the differentiation trees, which form the parametric potential reality defined by Alexander Gurwitsch [70] as a morphogenetic field and by Waddington [65] as the epigenetic landscape. Alicea et al. [71] discuss the mechanism that underlies the formation of differentiation trees and governs collective cell behaviors and the coordination of tissue development via interaction between the cytoskeletal dynamics and the genome. The relation of cytoskeletal differentiation waves [23,72] to the genome via the regulation of expression of the differentiation wave-related proteins could be achieved by the epigenome molecular mechanism. This mechanism provides fine control of gene expression through the direct interaction of the cytoskeleton with the network of transcription factors mediated by small RNAs.

The formation and functioning of the mitotic spindle represents the case of catalysis of cell reproduction by the cytoskeleton. Mitotic spindle orientation, controlled, in particular, by the actin cortex and influenced by cell geometry and mechanical forces, is essential for cell fate decisions, epithelial maintenance, and tissue morphogenesis [73]. While the mitotic process governs morphogenesis, a more complex process of the reduction of the number of chromosomes during the formation of germ cells in meiosis is involved in the governance of the process of evolution in eukaryotic organisms.

In the process of neural development, the coordinated operation of the cytoskeletal rearrangements and the small RNA action results in the growth and differentiation of the neural system. The experiments demonstrated that microtubules align the neuroepithelium during neural tube closure via their participation in interkinetic nuclear migration, leading to changes in cell shape [74]. The process of conversion of fibroblasts to neurons is mediated by microRNAs [75]. The action of microRNAs in the developing nervous system consists, in particular, of the regulation of subunit compositions of ATP-dependent chromatin remodeling complexes, which directly control neural development [76].

## 7. Emergence of Consciousness and Social Evolution: The Third Functional (Reflexive) Cycle

The complexification of multicellular eukaryotic organisms and their nervous system opened the possibility of the hierarchically highest level of reflexive activity that perceives and evaluates the preceding activity of the second functional cycle. While the second functional cycle is associated with the predetermined instinctive behavior, its perception in the third functional cycle generates a new field of meanings associated with conscious experience. The underlying physical process that provides a condition for reflexive conscious activity remains a matter of future investigation. Similarly to the second functional cycle and to the events occurring in the morphogenetic process, we can suggest that conscious activity is based on the perception and recognition of low-energy quanta emitted during the operation of the second loop of perception, which form the system of reflexive awareness that emerged through the evolution of the nervous system. These low-energy quanta bear information about the operation of the second loop and can be recognized by specific internal receptor systems. Revealing this mechanism would mean the realization of the program of Alexander Gurwitsch [53,70], which was formulated in its original form a century ago.

The emergence of the third functional cycle, with the appearance of consciousness that governs the operation of the second cycle via reflexive activity, follows from the model of consciousness introduced by Vladimir Lefebvre [77]. In the framework of this concept, the coordination of the loops of perception and their reflection assumes the existence of resonance between them. According to Petoukhov [78,79], this appears through coordinated oscillations. Resonance underlies the coordination of bioenergetics processes and metabolic cycles [80] and plays an important role in morphogenetic electric fields [56]. The fundamental challenge of biology is to define the resonances between the primary, secondary, and tertiary cycles of perception and information processing. The discovery of molecular receptors involved not only in the perception of external signals but also in the perception of weak electromagnetic signals produced by the primary and secondary loops in the course of the internal reflexive control of perception will be the basis of the understanding of the physical background processes that make possible the evolutionary emergence of consciousness. These processes determined the enormous expansion of semiotic meaningful information that characterizes the dynamics and evolution of human civilization.

## 8. Conclusions

We have presented the concept that expands the idea of the functional circle (*Funktionskreis*) developed by Jakob von Uexküll for the explanation of the phenomena of adaptation of biological systems to their environment via the perception of weak signals and the reaction on them. In brief, the concept adopts the existence of additional reflexive functional cycles extracting meaningful information not from the environment but from the organism performing the basic functional cycle. The phenomena of eukaryogenesis and multicellularity appeared through the development of the second functional cycle, which perceives the initial cycle and reacts to the changes in its operation. In the first eukaryotic organisms, this took place at the level of complex eukaryotic cells and the interaction between their organelles. In multicellular organisms, the coordinated regulation of their processes is governed via the set of functional cycles operating through the intercellular communication mediated by the nervous system in animals and other coordinating structures. The cytoskeletal fibrils expand the conformational relaxation of biological macromolecules to the level of the cell and then to the whole organism. At the higher level of organization, the ultimate reflexive loop of consciousness appears and becomes the premise for the infinite growth of the semiotic system related to social activity and exhibited in language, arts, etc.

The emergence of evolutionary innovations (genome change; the emergence of eukaryotes, multicellularity, nervous systems, and their perception-action joining; and, finally, consciousness) occurs via the codepoesis, i.e., the creation of new codes as organisms and their interactions with the world complexify over evolutionary time. New codes emerge when a system is faced with undecidability and incomplete information upon which to act, codifying that very situation itself, signifying it into a new coding element, and then assigning values to that newly codified and now fully meaning-bearing sign. Through the process of codepoiesis, the emerging new analogous signal transduction events forming the process of semiogenesis in reflexive functional cycles are memorized with the appearance of new digital coding systems (codepoiesis). The nucleotide sequences alone provide the formally incomplete information upon the achievement of the limits of adaptability, which induces the processes of searching for new genomic configurations via processes such as meiotic crossover, and the epigenetic codepoesis becomes a major driver of evolutionary change (Box 3). The principle of complexification represents a natural law, which establishes that complex entities are selected because they are richer in the information that enables them to perform new functions [81]. Through complexification, the system achieves the condition of maximization of its power via synergistic effects [82]. This corresponds to the property of goal-directedness via the internal ability of the system to self-organize and evolve toward the attractors formed within the autopoietic organization [83,84].

Box 3 **Combinatorial rearrangements generate new meaning-bearing features of biological systems and processes.**

Semiogenesis in functional cycles → Incomplete information in nucleotide sequences → Memorization of new reflexive loops via codepoiesis → Achievement of sustainable non-equilibrium state → Maximization of the system’s power

The process of generating the codes of language provides the basis for generating different sociotypes. It determines collective communication between the individuals forming social groups [85]. The emergence of novel evolutionary realizations in biological and social systems corresponds to an expansion of reality in the potential form representing the meaningful information inherent to biological and social systems [64]. Any novel evolutionary feature obtains its semiotic value if it receives a particular interpretation simultaneously expressed as a meaningful function. The evolutionary growth of meanings in biological and social systems embedded in their environment generates the dynamics of the growing semiosphere formed and interpreted by the organisms inhabiting it. With the emergence of reflexive conscious activity, further expansion of the field of meanings takes place, forming the noosphere constituent of the biosphere. It represents the field of meanings generated by the reflexive loop of consciousness that produces such different semiotic structures as language, formal logic and mathematics, and the arts.

## Data Availability

No data were generated for this study.
